# BRIDGING THE GAP BETWEEN ACADEMIA AND PRACTICE: THE DUTCH LIVING LAB IN AGEING AND LONG-TERM CARE

**DOI:** 10.1093/geroni/igae098.0715

**Published:** 2024-12-31

**Authors:** Jan Hamers, Hilde Verbeek

**Affiliations:** Maastricht University, Maastricht, Limburg, Netherlands; Maastricht University, Maastricht, Limburg, Netherlands

## Abstract

There is a strong need in long-term care for scientific research, so older people and their families, health care professionals, policy makers, and educators can benefit from new advancements and best available evidence in everyday care practice. The model of a sustainable and successful interdisciplinary collaboration between scientists, care providers and educators in long-term care will be presented: the ‘Limburg Living Lab in Ageing and Long-Term Care’. It is a structural collaboration between academia, educational institutions, long-term care providers and clients. It covers approximately 185 long-term care facilities (e.g., nursing homes, assisted and group living facilities) as well as professional home care, and includes about 50,000 clients and more than 27,000 staff. Their mission is to contribute with scientific research to improving i) quality of life of older people and their families; ii) quality of care and iii) quality of work of those working in long-term care. Key working mechanisms are the scientific and practice-based Linking Pins and interdisciplinary partnership using a team science approach, with great scientific and societal impact. For more than 25 years this structural collaboration has served as an infrastructure that drives scientific research in long-term care in co-creation with end-users, including older people and their relatives, health care professionals, policy makers and educators. This model was established by Maastricht University in the Netherlands, and more recently, the model of the living lab is replicated in other countries such as the United Kingdom, Germany and Austria.

